# Seeing is believing

**DOI:** 10.7554/eLife.21583

**Published:** 2016-10-25

**Authors:** Enrique Amaya

**Affiliations:** Faculty of Biology, Medicine and Health, University of Manchester, Manchester, United Kingdomenrique.amaya@manchester.ac.uk

**Keywords:** regeneration, live imaging, *Parhyale hawaiensis*, Other

## Abstract

A small transparent crustacean called *Parhyale hawaiensis* has become a powerful model system for the study of limb and appendage regeneration.

**Related research article** Alwes F, Enjolras C, Averof M. 2016. Live imaging reveals the progenitors and cell dynamics of limb regeneration. *eLife* **5**:e19766. doi: 10.7554/eLife.19766

There is little that fires the imagination like the possibility of regrowing body parts following an injury. Indeed the capacity to regrow organs and limbs – and heads and tails – is the stuff of myths and legends dating back to antiquity. And as remarkable and magical as it may appear, the ability to regrow lost or injured body parts is a reality for a significant proportion of the animal and plant kingdoms. Indeed, it is possible to regenerate an entire plant from a small fragment of plant tissue or even from a single cell (Ikeuchi et al., 2016). And some animals – such as hydra, planarians and colonial ascidians – are also able to reproduce by regenerating an entire organism from part of its parent (Sánchez Alvarado, 2000).

Given that humans are not amongst those animals that can regenerate a limb or other body parts (never mind an entire organism) following an injury, there has been great interest in understanding the mechanisms employed by those animals that are capable of complex tissue regeneration. The ultimate hope is that this knowledge could lead to breakthroughs in regenerative medicine, including the regeneration of human tissues and organs (Brockes and Kumar, 2005).

Studying the molecular and cellular mechanisms of limb regeneration presents many challenges. First, limb regeneration takes a long time to complete: for example, in urodele amphibians (newts and salamanders) it typically takes about a month for larval stages and almost a year in some adult organisms (Young et al., 1983). Second, the model systems that have been used to study limb regeneration tend to take a relatively long time to reproduce (with generation times typically being 1–2 years) and to be relatively recalcitrant to genetic manipulation. Third, limb regeneration has largely been studied in systems that are opaque to imaging, so its dynamic nature has remained largely hidden behind closed doors. While new genomic and genetic tools for amphibians have become available in recent years (Hayashi et al., 2014; Khattak et al., 2013; Flowers et al., 2014), the long generation times and the opaque nature of these organisms remain an obstacle. Now, in eLife, Frederike Alwes, Camille Enjolras and Michalis Averof of the École Normale Supérieure de Lyon report how a small crustacean called *Parhyale hawaiensis* offers a solution to all of these problems (Alwes et al., 2016).

The arthropods represent the largest phylum in the animal kingdom and representatives of this phylum – notably the fruit fly, *Drosophila melanogaster* – have long been used to study development, genetics and evolution. Unfortunately for researchers, fruit flies cannot regenerate their limbs. However, *Parhyale hawaiensis* retains many of the advantages of *Drosophila* (a relatively short generation time, the transparency of its embryos and adults, and the availability of advanced transgenic tools) and it is also able to regenerate its appendages (antennae, mouth parts and limbs) within a week as an adult (Konstantinides and Averof, 2014; Grillo et al., 2016). In particular, rapid advances in recent years mean that it is now possible to manipulate the genome of *Parhyale hawaiensis*, through transgenesis and targeted gene modification approaches, with relative ease (Stamataki and Pavlopoulos, 2016).

The final challenge, as far as the study of limb and appendage regeneration is concerned, is to image the entire regeneration process at the single cell level in adult animals. This is particularly challenging as adult animals like to move. One approach would be to anaesthetize the organism while imaging: however, it takes a number of days to complete the regeneration processes, and few animals can survive being anaesthetized for this length of time. Alwes, Enjolras and Averof overcame this problem in *Parhyale hawaiensis* by gluing one of its legs to a cover slip, which allowed the rest of the animal to continue to move and feed. Next they amputated the immobilized leg, which then proceeded to regenerate (without moving) inside the transparent exoskeleton of the leg. In the next molt, the animal freed itself from the glued exoskeleton (which remained behind) and emerged with a new, fully functional, leg. This new approach allowed Alwes et al. to follow the dynamic nature of appendage regeneration at the single-cell level over a period of 4–5 days (Figure 1). This is the first time that this has been done in any organism.

**Figure 1.**
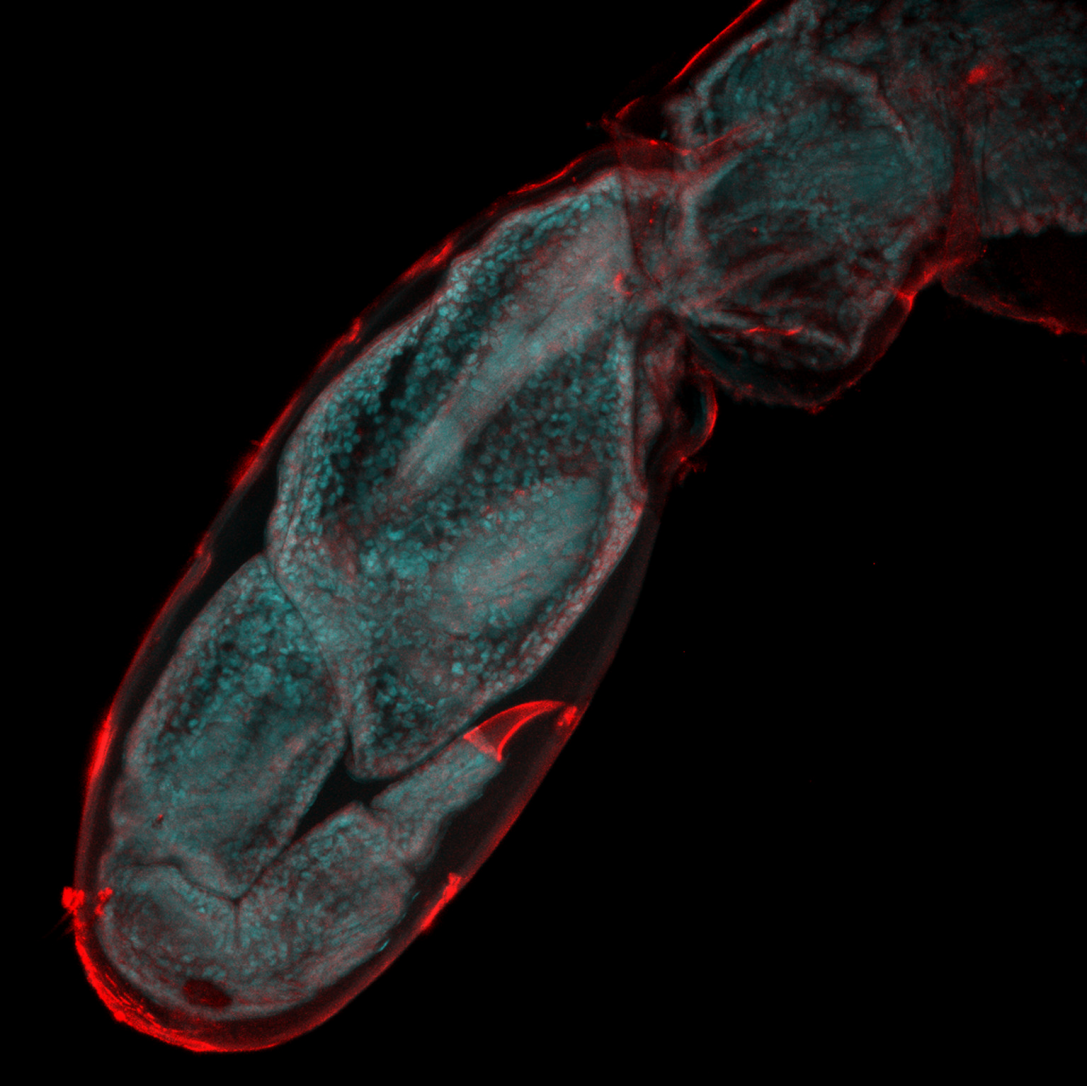
Watching limb regeneration in *Parhyale hawaiensis*. Alwes et al. amputated a leg (called the T5 limb) in the crustacean *Parhyale hawaiensis*, and then used a combination of different microscopy techniques to follow its regeneration. This image, taken six days after amputation, shows the regenerated limb encapsulated within the cuticle of the previously amputated limb (red); the image is 655 microns across. Image provided by Frederike Alwes.

The ability to visualize record the dynamic nature of limb regeneration at the cellular level, combined with the availability of a number of genetic approaches, means that many of the secrets underlying appendage regeneration will likely be finally revealed using this humble, but beautiful, little crustacean.
